# High Patient Willingness to Grant Broad Consent for Real-World Data Use in Rheumatology—Implications for Real-World Data Platform Governance: Cross-Sectional Study

**DOI:** 10.2196/80347

**Published:** 2026-06-08

**Authors:** Jutta G Richter, Antonia Becker, Tim Filla, Hasan Acar, Waldemar Ockert, Dominykas Kriauciunas, Edith Aggarwal, Markus Schröder, Ralf Hansen, Jörg HW Distler, Matthias Schneider

**Affiliations:** 1 Department for Rheumatology, University Hospital Düsseldorf, Medical Faculty of Heinrich-Heine-University, Moorenstrasse 5, Düsseldorf, 40225, Germany; 2 Hiller Research Center, University Hospital Düsseldorf, Medical Faculty of Heinrich-Heine-University, Düsseldorf, 40225, Germany; 3 ZS Inc, London, United Kingdom; 4 Serrala Group GmbH, Berlin, Germany

**Keywords:** informed broad consent, secondary use, real-world data, governance, rheumatology, artificial intelligence, AI

## Abstract

**Background:**

Medical real-world data (RWD) are often siloed across organizations, making them inaccessible for research. Unlocking these data could advance clinical research and patient care. The pan-European Data Nexus platform (DNP) links RWD, facilitating its use, for example by artificial intelligence (AI) tools, to support the generation of real-world evidence. In Europe, particularly Germany, the secondary use of health data is governed by stringent regulatory requirements, including informed consent.

**Objective:**

This study evaluated the informed broad consent form for the RWD (Data Nexus) platform, predicated on the principles of the Medical Informatics Initiative in Germany, and contextualized its implications for future data governance and regulatory use of RWD.

**Methods:**

The broad consent form was developed for the DNP and cross-sectionally distributed to consecutive rheumatology outpatients during routine follow-up at a tertiary center. Analyses included rates of agreement to the predefined broad consent items. A zero-inflated model (using R) was used to predict response rates.

**Results:**

From July 2023 to May 2024, 74.9% (292/390) of the patients signed the broad consent form and consented to DNP data donation. Median age was 56 (IQR 43.0-65.0) years, 72.5% (211/291) were female, and median disease duration was 12 (IQR 4.0-22.0) years. Diagnoses included rheumatoid arthritis (96/291, 33.3%), psoriatic arthritis (30/291, 10.3%), spondyloarthritis (15/291, 5.2%), systemic lupus erythematosus (90/291, 30.9%), systemic sclerosis (14/291, 4.8%), and other conditions (16/291, 5.5%). Patients also answered 8 yes/no broad consent items, with an average of “yes” and “no” responses of 7.5 (SD 1.5) and 0.2 (SD 0.5), respectively. Missing responses averaged 0.4 (SD 1.4). Of all participants, 78.4% (228/291) agreed to all broad consent items. Approval rates for individual items exceeded 86%, indicating strong patient acceptance of secondary use of RWD under a structured governance framework, possibly reflecting trust, perceived benefit, low perceived risk, and governance confidence. Importantly, patients agreed to new techniques such as AI-based analysis of their donated RWD and, despite the social and ethical sensitivity, data distribution to third parties, including commercial industry. Consent rates were also high for the use of valuable omics and genomics data from biomaterial donations. Women showed higher consent rates, whereas educational attainment was not an indicator of response behavior.

**Conclusions:**

This is the first study assessing the willingness of patients with inflammatory rheumatic diseases to grant broad consent for secondary data use in an innovative RWD platform, implementing a modern framework in routine care and considering detailed patient preferences. Patients demonstrated high willingness to grant broad consent, providing key real-world evidence on patients’ actual consent behavior for implementing RWD platform integration—such as the European Health Data Space. The DNP supports scalable, General Data Protection Regulation–compliant data sharing, enabling real-world and AI-driven research while preserving patient trust.

## Introduction

Health care data captured during routine clinical care are typically scattered across various institutions and their systems. Real-world data (RWD; eg, from clinics, private practices, and patients) encompass information from experiences and observations of individuals, organizations, and systems. Unlike randomized controlled trials (RCTs), which have strict inclusion and exclusion criteria, RWD reflect the complexities and nuances of real life, offering rich medical insights [1].

Traditionally, RWD sources (eg, claims data, electronic medical records [EMRs], and registries) have been used for epidemiological and health economics research. Their research utility can be enhanced through cross-source linkages for holistic views of patients’ health and experiences. The rigorous level of data control in RCTs makes them the gold standard for effectiveness evidence to establish treatment efficacy and safety, and RWD acceptance for these purposes has been limited [2-5]. However, RWD play a key role in closing information gaps from RCTs to generate real-world evidence (RWE) on outcomes in diverse populations receiving routine care [6]. RWD are increasingly regarded as a critical resource (eg, for clinical research, artificial intelligence [AI]–enabled research, regulatory decision-making, and health technology assessment [HTA]), and their use is gaining acceptance in research and regulatory decision-making and generating major interest in progressing frameworks [7-10].

The management of RWD within modern IT infrastructure and techniques is evolving. Collecting and analyzing RWD from various sources currently not fully leveraged for secondary research purposes requires an adequate, robust IT infrastructure and advanced methods such as AI [1,10]. The emergence of European data spaces, specifically, secure processing environments (SPEs; virtual environments for secure data storage, management, and analysis), highlights the importance of leveraging RWD with robust governance [11,12]. Regulatory bodies and networks aim to harmonize the collection of high-quality RWD and reliable RWE generation (eg, for HTA documents [10,13-15]). RWD and RWE may influence and improve national and international health policy [16]. Successful examples of RWD for rheumatology research include the EMR-enabled Rheumatology Informatics System for Effectiveness registry and the PremiOM psoriatic arthritis (PsA) dataset [17,18]. The Rheumatology Informatics System for Effectiveness automatically extracts structured data from routine clinical care for research and quality improvement, and PremiOM combines claims and EMR data at the individual level to study real-world treatment patterns [17,18].

In Europe, initiatives such as the European Health Data Space (EHDS) aim to facilitate the secondary use of health data across borders, including for research and innovation purposes [19,20]. Within this evolving landscape, broad consent has emerged as a key ethical and legal mechanism to enable the future use of RWD and biospecimens, specifically their related data for RWE generation via emerging AI techniques [21]. However, broad consent’s acceptance among patients and the factors influencing consent decisions remain important considerations, particularly in light of ongoing debates on data governance, privacy, and trust.

Patient consent is fundamental to respecting patient autonomy and sustaining trust in health care and health-related research [22]. Moreover, both German legislation and the European General Data Protection Regulation (GDPR) mandate informed consent for the secondary use and reuse of RWD. As RWD-driven research infrastructures and SPEs gain strategic importance for data-driven health care and AI research, empirical evidence on patients’ willingness to share health data remains limited, particularly among individuals with inflammatory rheumatic diseases (IRDs) [23,24].

To address this gap, this study is the first to systematically evaluate the willingness of patients with inflammatory rheumatic diseases (IRDs) to grant broad consent for secondary data use within an SPE. The primary objective is to assess the feasibility and acceptance of collecting and reusing patient-generated RWD within the pan-European Data Nexus platform (DNP) while deriving implications for the implementation, governance, and scalability of emerging European SPE initiatives, including the EHDS.

## Methods

### Overview of the Platform and Study Design

**Figure 1.**
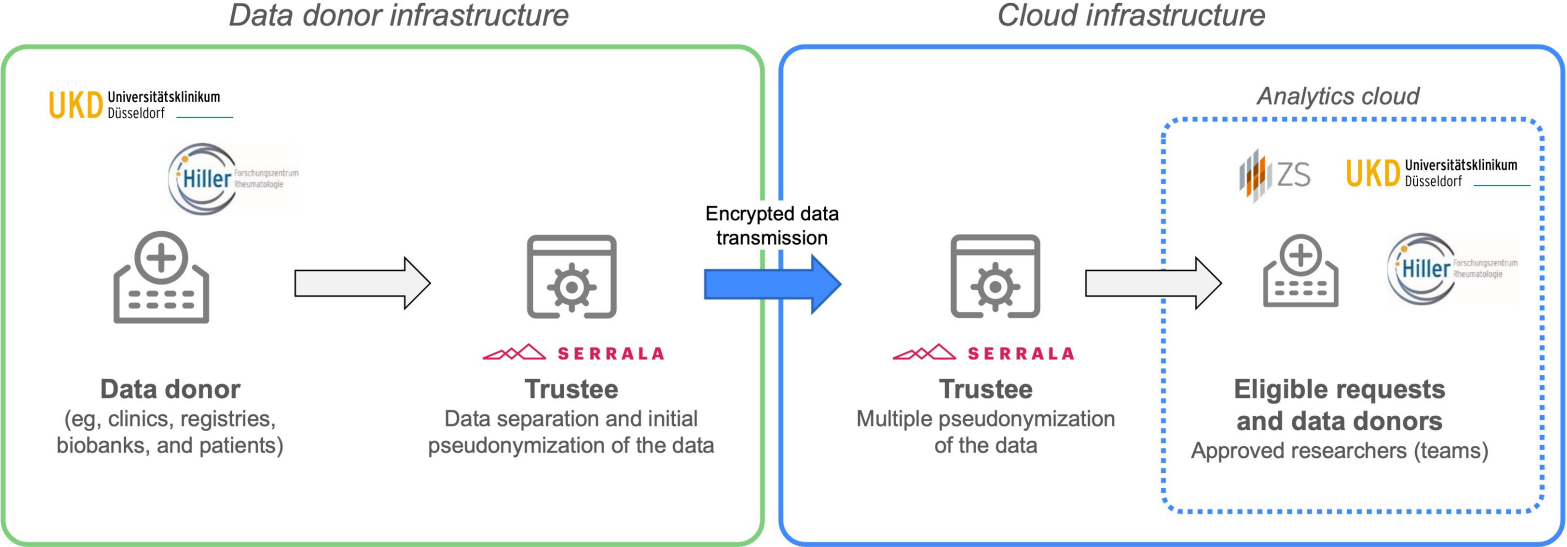
Outline of the Data Nexus architecture depicting data flow and processing.

The DNP supports medical research and health care by integrating RWD in a secure environment. The DNP aims to optimize early detection, treatment, and prevention of diseases through insights from RWD research. Figure 1 depicts the DNP’s scope and remits. The consortium implemented a comprehensive data management framework and a use and access committee to oversee authorized, eligible data requests and analyses pursuant to the broad consent forms.

**Figure 2.**
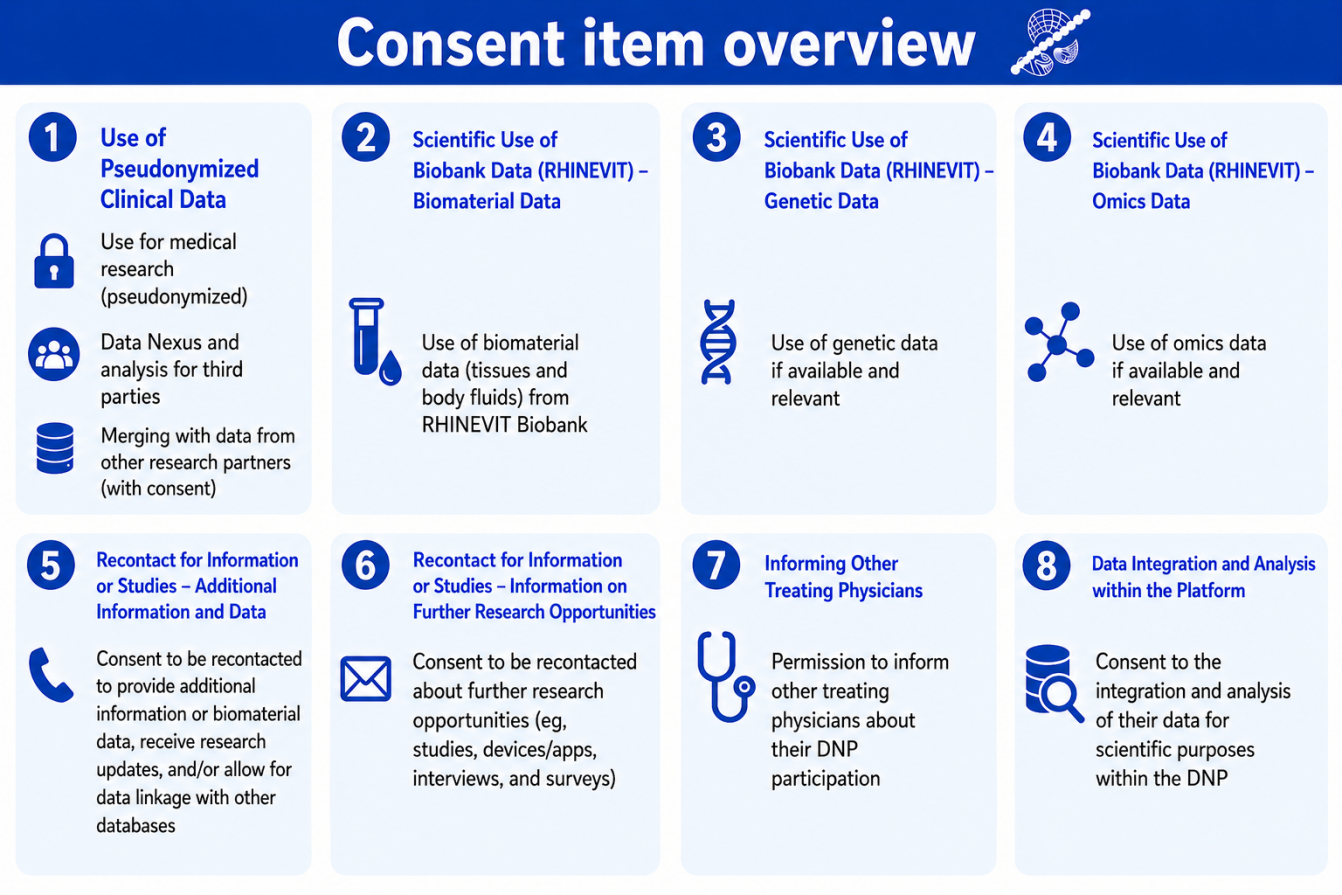
Graphical representation of the 8 Data Nexus platform broad consent items.

To enable research within the DNP, the informed broad consent form of the German Medical Informatics Initiative (MII) was adapted [25]. The broad consent form, including additions made by us, was approved by the responsible data protection officer and ethics committee. Details on the items’ wording are outlined in the Results section and depicted in Figure 2. Patients consented to the transfer of existing data (including clinical and sociodemographic data) and prospectively generated data (eg, disease information, treatment, and patient-reported outcomes from routine care after consent) for the following 5 years. After 5 years, individual broad consent must be renewed and processed accordingly. Withdrawal procedures were described in the patient information section of the broad consent form.

Patients could agree to or decline 8 individual broad consent items with either a “yes” or “no” response. If no choice was made, the answer was classified as missing, handled as “not given” in the DNP. Data were processed only for signed broad consent forms and solely according to the items that indicated “yes.”

RWD parameters originated from our patient documentation system DocuMed.rh, which also holds data from the local electronic health record (EHR) [26]. According to patients’ broad consent, biomaterial data (including genetic and omics data) could be used for research from the clinic’s biobank RHINEVIT [27]. Data from office-based physician practices or other resources can be integrated into the DNP.

### Study Center

We piloted this platform and the broad consent form in a tertiary rheumatology center at a German university clinic to evaluate the feasibility of collecting patient data using a platform such as the DNP. To this end, we systematically assessed and reported our observations with the aim to demonstrate strong patient acceptance of broad consent, which is highly relevant for the future of RWE generation and data-driven research infrastructures.

### Patient Characteristics and Inclusion and Exclusion Criteria

Patients with IRDs coded in the *International Classification of Diseases, 10th Revision*, as rheumatoid arthritis (RA), PsA, spondyloarthritis (SpA), systemic lupus erythematosus (SLE), systemic sclerosis, vasculitis, and others above the age of 18 years were included randomly. Adequate German-language proficiency was considered a prerequisite. Exclusion criteria comprised age of less than 18 years and insufficient language proficiency to ensure adequate comprehension of broad consent and well-informed consent. Thus, we included 291 patients (n=211, 72.5% female).

### Data Collection Processes

Consecutive patients with IRDs attending routine outpatient follow-ups in our rheumatology clinic received paper-based information about the DNP and the broad consent document during in-person visits. The 8 broad consent items were always listed in the same order, each requiring a “yes” or “no” response, although they could be left unanswered. A DNP team member from the rheumatology department explained the DNP face-to-face as in-person interaction is considered most effective for improving participants’ understanding of consent forms [28]. Afterward, patients read the patient information and provided responses in the waiting or treatment room. The DNP team member remained available for content-related or technical questions. If consent was given, 1 signed copy of the broad consent form was returned to the DNP team, and another was handed out for the patients’ personal records.

Signed paper-based forms were entered manually into a database. To minimize data entry errors, 2-pass verification was performed. Digitized broad consent forms were uploaded to the DNP for informed consent–compliant data handling within the DNP. Paper-based forms were stored securely in the clinic following German medicolegal requirements.

For quality assurance purposes, data from the broad consent forms and related (clinical) data from the period from July 2023 to May 2024 were extracted. We analyzed the consent rates of the signed broad consent forms for each of the 8 broad consent items.

### Measures and Covariates

Patient acceptance of the predefined 8-item broad consent form was measured by analyzing responses to each consent item, allowing for both overall acceptance and item-specific variation. Eight predefined broad consent items could be answered with “yes” or “no” or left unanswered. For data quality assurance, we extracted age, sex, clinical data, and patients’ educational level, assessed in our patient documentation system, where the data are documented in alignment with the standards of the national database [26,29]. The broad consent form allows for the analysis of the data. Due to resource constraints, the sample size was limited, and a qualitative approach was not followed.

### Ethical Considerations

The DNP broad consent form received a positive vote from a local independent ethics committee (ethics committee of the Medical Faculty of the Heinrich-Heine-University Duesseldorf, 2022-1958). The DNP is registered in the German Clinical Trials Register (DRKS00032801). This study was conducted in accordance with ethical principles and applicable institutional and national regulations. Participants provided written informed consent. No financial or other compensation was provided to patients for study participation. All procedures were carried out in accordance with the Declaration of Helsinki. To safeguard the privacy and confidentiality of human participants, all data are presented in an aggregated and nonidentifiable format. No identification of individual participants in the manuscript or supplementary material is possible.

### Sample Size and Statistical Analyses

Due to the exploratory design of this study, no sample size calculation was performed. Descriptive statistics are provided as absolute numbers and percentages for discrete variables and as medians and IQRs for continuous variables. For statistical analysis of multi-class variables, a chi-square test was used. In addition, one-way ANOVA was calculated where appropriate according to the variable level. It was assumed that there were 2 types of patients: one group agreeing to all items without reading them in too much detail and a second group of patients who checked each item or at least some items individually on a more detailed level. The chosen zero-inflated model captures a continuum of patient behavior between these 2 extremes. To determine which factors predict patients’ agreement with the broad consent items, 2 zero-inflated models were calculated using age, sex, educational level, disease, and disease duration as the independent variables and the number of nonconsented or missing items as the dependent variable. Imputation of missing values was not conducted because missing values were regarded as 1 of 3 possible item outcomes (“yes,” “no,” and left unanswered). The zero-inflated model accounts for heterogeneity in response patterns, including the possibility that a subset of respondents will exhibit consistently high acceptance across items, which may reflect differences in engagement, decision strategies, or response behavior. Furthermore, a sensitivity analysis for the risk factor analysis of patients’ consent was conducted using a logistic regression approach with a binary outcome of “consented all” (yes or no) as the dependent variable and the same set of independent variables as for the zero-inflated model. A Little missing completely at random test was performed. A *P* value of less than .05 was considered statistically significant. All statistical computations were conducted using R (version 4.3.1; R Foundation for Statistical Computing). Data were analyzed anonymously.

## Results

### Overview

**Figure 3.**
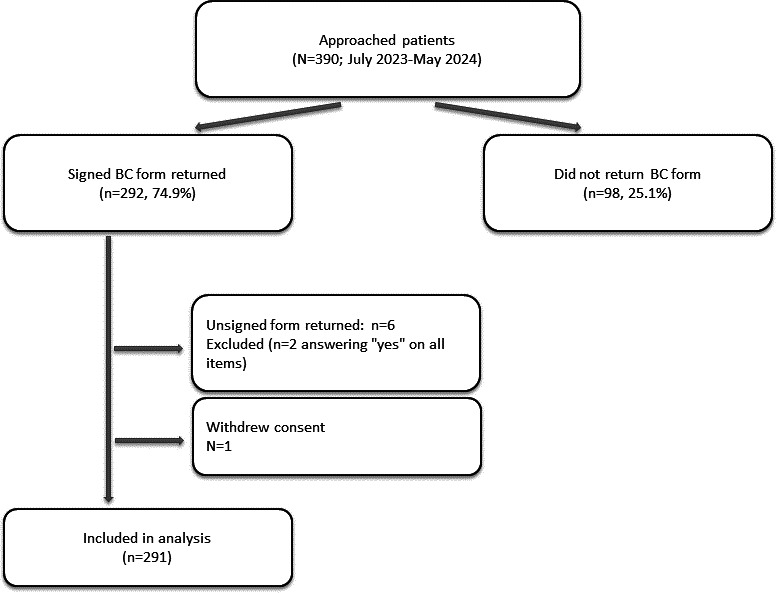
Participant flowchart. BC: broad consent.

Of 390 consecutive outpatients receiving the broad consent form between July 2023 and May 2024, a total of 292 (74.9%) returned it signed. Thus, they provided voluntary broad consent for a period of 5 years from the date of consent and consented to the extended data use beyond this period (30 years, as outlined in the patient information). Refusal was not attributable to the content of the patient information or broad consent form as none of the individuals at the clinic had reviewed the document. Documented reasons for denial included time constraints, general lack of interest in research participation, and failure to return the document after taking it home. Of the 292 patients who signed the broad consent form, 1 (0.3%) male patient withdrew his consent 5 months after signing. Thus, further analyses refer to 291 patients. A total of 1.5% (6/390) of the broad consent forms were returned unsigned and, therefore, excluded from analysis; 2 of these had selected “yes” for all broad consent items. Participant flow is shown in Figure 3.

Content-related or technical questions while or after reading the patient information and the broad consent form were scarce (2/291, <1%) and questions from others related exclusively to which hard copies could be kept and which must be returned. Team members spent up to 15 minutes talking one-on-one to individual outpatients.

Participants’ median age was 56 (IQR 43.0-65.0) years, and most were female (211/291, 72.5%). Median disease duration was 12 (IQR 4.0-22.0) years. Further clinical and sociodemographic characteristics of the cohort are shown in Table 1.

**Table 1.** Clinical and sociodemographic characteristics of the complete group and distinct disease subgroups (N=291).

	All	RA^a^ (n=96)	PsA^b^ (n=30)	SpA^c^ (n=15)	SLE^d^ (n=90)	SSc^e^ (n=14)	Vasculitis (n=30)	Others (n=16)
Female sex, n (%)	211 (72.5)	63 (65.6)	17 (56.7)	8 (53.3)	80 (88.9)	12 (85.7)	21 (70)	10 (62.5)
Age (y), median (IQR)	56 (43.0-65.0)	58 (46.3-66.0)	53 (30.8-64.3)	58 (51.0-61.0)	49 (40.8-58.0)	52 (44.8-59.8)	66 (57.0-78.0)	60 (43.5-66.8)
Disease duration (y), median (IQR)	12 (4.0-22.0)	9.0 (3.0-17.5)	9.0 (1.8-17.8)	9.0 (4.0-18.0)	21.0 (13.0-27.0)	8.5 (1.0-15.5)	6.0 (2.0-12.3)	10.0 (1.3-20.0)
University entrance diploma,^f^ n (%)	114 (48.5)	34 (45.3)	12 (54.5)	5 (41.7)	42 (51.9)	6 (60.0)	11 (44.0)	4 (36.4)

a RA: rheumatoid arthritis.

b PsA: psoriatic arthritis.

c SpA: spondyloarthritis.

d SLE: systemic lupus erythematosus.

e SSc: systemic sclerosis.

f In Germany, this is a secondary school diploma that permits entrance to university. Not all patients reported their educational level, so the percentages may not match the denominators in the column headings.

The average number of “yes,” “no,” and missing responses was 7.5 (SD 1.5; range 0-8), 0.2 (SD 0.5; range 0-3), and 0.4 (SD 1.4; range 0-8), respectively. No relevant differences in the number of “yes,” “no,” and missing responses were noted between disease or educational level groups. Female patients gave statistically significantly more “yes” responses compared to male patients (mean 7.6, SD 1.2 vs mean 7.1, SD 1.9; *P*=.008) and had significantly fewer missing responses (mean 0.3, SD 1.1 vs mean 0.7, SD 1.9; *P*=.01). “No” responses were similar between female and male patients (mean 0.2, SD 0.5 for both; *P*=.45). A total of 2.1% (6/291) of the patients with signed the broad consent forms did not select any of the 8 broad consent options and showed similar characteristics to those of the overall population (50% female; median age 56 (IQR 43-65) years; median disease duration 12 (IQR 4-22) years; 40% with a secondary school diploma granting university entrance; n=2, 33.3% with RA; n=1, 16.7% with PsA; n=1, 16.7% with SpA; n=1, 16.7% with SLE; and n=1, 16.7% with other diseases).

**Figure 4.**
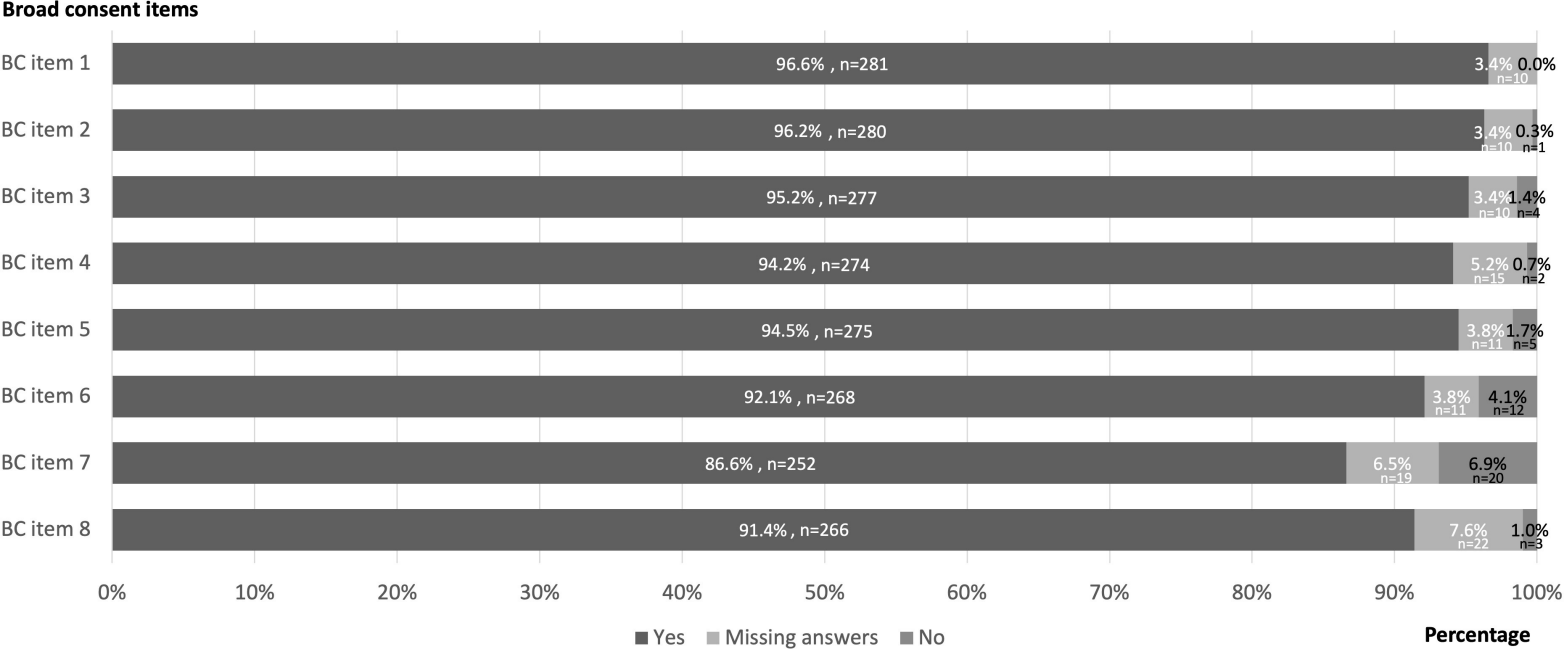
Response rates (“yes,” “no,” and missing) for the 8 broad consent (BC) items.

Overall, approval rates for the 8 broad consent items exceeded 86% (Figure 4). Detailed results for each item are presented below.

### DNP Broad Consent Item 1

Item 1 covered 3 domains. First, it asked for permission to process and use patient data for medical research as described in the patient information, ensuring multi-pseudonymization. Data would be derived from our rheumatology-specific patient documentation system. Second, it covered scientific analysis and use of the data by the DNP and, thus, by extension, for third parties (eg, universities, institutes, and research companies [pharmaceutical companies and medical device manufacturers]). This may also include remote access to data for research projects conducted abroad in places where European data protection law applies. Patients were informed that they would not benefit commercially. Third, it asked for permission to merge their patient data with other research partners’ data if the patients agreed to this use by those partners. A total of 96.6% (281/291) of the patients consented to this option, none declined, and 3.4% (10/291) gave no answer.

### DNP Broad Consent Item 2

Patients were asked whether biomaterial-linked data from the clinic-owned RHINEVIT biobank could be transferred and used within the DNP if available from prior donations. A total of 3.4% (10/291) of the patients gave no answer, 96.2% (280/291) consented, and 0.3% (1/291) declined.

### DNP Broad Consent Item 3

If genetic data were available from the clinic’s biobank RHINEVIT, specifically, our patient documentation system DocuMed.rh, and if these were relevant for their IRD, the patients’ comorbidities, and research, 95.2% (277/291) of the patients consented to their scientific use as outlined in the patient information. In total, 1.4% (4/291) of the patients declined, and 3.4% (10/291) did not answer.

### DNP Broad Consent Item 4

Correspondingly, if omics data were available that were related to the IRD, specifically, associated comorbidities, 94.2% (274/291) of the patients agreed to their scientific use, whereas 5.2% (15/291) provided no answer, and 0.7% (2/291) declined.

### DNP Broad Consent Item 5

Consent was given for contacting the patients to obtain additional information, use additional RHINEVIT biomaterial-related data, provide updates and information on research projects, and link patient data with other medical databases by 94.5% (275/291) of the patients, whereas 1.7% (5/291) declined, and 3.8% (11/291) provided no answer.

### DNP Broad Consent Item 6

Similarly, 92.1% (268/291) of the patients consented to being contacted about future research opportunities that may be relevant to them, 4.1% (12/291) declined, and 3.8% (11/291) did not answer. Common reasons for conducting research included but were not limited to assisting in the research of new medical devices and digital health applications, learning more about clinical trials and participating in trials relevant to them, and engaging in DNP-related research (eg, interviews and surveys).

### DNP Broad Consent Item 7

Permission to inform other treating physicians about their DNP participation was granted by 86.6% (252/291) of the patients. Interestingly, 6.9% (20/291) declined, and 6.5% (19/291) did not answer. The decliners were mostly female (14/20, 70%), with a median age of 45 years and a median disease duration of 12 years; 50% (10/20) held a university entrance diploma. The diseases experienced by those who declined included RA (3/20, 15%), PsA (4/20, 20%), SpA (2/20, 10%), SLE (8/20, 40%), vasculitis (1/20, 5%), and others (2/20, 10%).

### DNP Broad Consent Item 8

Most patients (266/291, 91.4%) consented to the integration and analysis of their data for scientific purposes within the DNP, whereas 7.6% (22/291) did not answer. A total of 1.0% (3/291) of the patients declined (66.7% female; median age 45 years; median disease duration 12 years; all university entrance diploma holders; and 1 each with a diagnosis of RA, SpA, and SLE). Both of the female patients who declined also declined to inform other treating physicians about their DNP participation.

### Information Material Left Behind at the Clinic

A minority of patients (33/291, 11.3%) left the information material behind at the clinic; 88.7% (258/291) took the material with them. A lower percentage of women (19/211, 9%) than men (14/80, 17.5%) left the material behind. Those who left it were younger (median age 51 vs. 56 years of age) and had a longer disease duration (median 15.0 vs. 12.0 years). Patients of all disease groups (range 1/291, 3% [SpA] to 11/291, 33.3% [RA]) left the material at the clinic. The educational level of these patients was similar: 48% (12/25) of those who left the material at the clinic and 48.6% (102/210) of those who took it with them had a university entrance diploma. Missing data for educational level taken from RWD comprised 19.2% (26/291).

### Results From the Zero-Inflated Model

We investigated a zero-inflated model to detect which variables predicted patients’ agreement to the broad consent form items. The model could detect sex (female) but no other determinants of response behavior (Table 2). As already shown above, female patients had a much smaller number of missing items, clearly pointing to a systematic pattern rather than a random one, which can be regarded as a proxy for better reading of the broad consent form compared to male patients. The Little missing completely at random test was not significant (*χ*^2^_81_=92.4; *P*=.18), indicating that missing data were likely missing completely at random.

**Table 2.** Results of the zero-inflated model.

Predictor	Number of “no” responses		Number of missing responses	
	Incidence rate or odds ratio (95% CI)	*P* value	Incidence rate or odds ratio (95% CI)	*P* value
Age (y)	1.01 (0.97‐1.05)^a^	.67	0.99 (0.97‐1.00)^a^	.15
Disease duration (y)	0.99 (0.94‐1.03)^a^	.51	0.99 (0.96‐1.02)^a^	.38
Educational level	1.17 (0.33‐4.10)^a^	.81	0.89 (0.53‐1.47)^a^	.64
Gender (female)	2.07 (0.54‐7.89)^a^	.29	1.12 (0.64‐1.94)^a^	.70
Zero-inflated model				
Age (y)	1.02 (0.97‐1.07)^b^	.40	0.98 (0.95‐1.01)^b^	.11
Disease duration (y)	0.98 (0.91‐1.04)^b^	.46	0.99 (0.96‐1.03)^b^	.70
Educational level	1.06 (0.26‐4.36)^b^	.93	0.86 (0.38‐1.94)^b^	.72
Gender (female)	4.58 (0.59‐35.57)^b^	.15	2.69 (1.15‐6.32)^b^	.02

a Incidence rates.

b Odds ratios.

## Discussion

### Principal Findings

This study represents the first investigation in rheumatology focusing on broad consent for an innovative, centralized, pan-European, cross-border research platform integrating RWD from multiple sources. With broad consent, the DNP provides secure access to multi-pseudonymized RWD for approved scientific researchers and Data Nexus analysts (eg, biostatisticians and epidemiologists) on behalf of pharmaceutical clients and medical device manufacturers. Importantly, our findings should be interpreted in the broader context of evolving data governance and regulatory science in Europe. While high consent rates demonstrate feasibility at the patient level, their full significance lies in enabling scalable infrastructures for secondary use of RWD in regulatory decision-making, HTA, and cross-border research initiatives such as the EHDS [10,13-15,20,21]. In this context, granular broad consent models such as the one implemented in the DNP may represent a foundational governance layer for interoperable, ethically compliant RWD ecosystems, bridging clinical care and secondary use across jurisdictions. Our data are unique to rheumatology and IRDs and can help address key knowledge gaps and support patient readiness to share data for complex use.

Our cohort reflects a broad IRD population, including various inflammatory diseases. A median disease duration of 12 (IQR 4.0-22.0) years indicated long-term disease involvement. Sex distribution aligned with IRD patterns, although SpA showed a slight female predominance in a small subgroup.

We observed an unexpectedly high approval rate despite no concrete benefits or incentives. This is comparable to approval rate data in oncology and higher than the approval rate observed in MII broad consent form data from an emergency department [30,31]. Recently, varying approval rates have been reported from university clinics [21]. The broad consent aspects were well accepted by our patients, with high consent rates to individual items and most consenting to all items. Similar to our previous biobank broad consent analyses, not all patients answered every item [27]. Unlike in those previous analyses, women in this study left fewer items unanswered, suggesting better broad consent form reading and greater willingness to donate data to the DNP [27]. As reported by others focusing on data donation from EHRs in Germany, educational attainment was not an indicator of response behavior in this study [24]. This contrasts with our own research on voluntary broad consent related to biomaterial donation, where higher-educated patients responded negatively to some broad consent items more often [27]. While age-dependent data donation willingness has been reported, age did not influence our patients’ consent rates [32].

In a representative German online population, positive attitudes toward data donation were influenced by clear distinctions between public and private research and strong trust in data protection and control [23]. Our broad consent form’s first item, which combines public and private research, achieved very high agreement rates. This indicates that patients are willing to donate data for scientific and clinical research purposes, and their willingness is not compromised when research is conducted on behalf of third parties. A recent systematic review confirmed a positive public attitude toward sharing personal health data for third-party or secondary use, albeit under certain conditions [33]. However, willingness to donate self-generated health data (eg, from wearables) for secondary use can vary by disease and country [34,35]. Our data align with those reported for German patients with cancer, where 73.9% supported RWD use by researchers in commercial companies [22]. A Bitkom (Germany’s digital association) survey also showed that a large majority of German citizens (90%) would share personal health data for research, including by private companies [36].

In this regard, the inclusion of commercial research within our broad consent frameworks requires particular (ethical) attention. While acceptance rates were high, transparency regarding the role of industry partners, data use pathways, and safeguards against misuse is essential to sustain patients’ and data providers’ trust. Public research institutions generally elicit a high level of trust in data use, whereas skepticism remains toward data use by private sector entities, particularly with regard to commercial motives and data security [22,32,37-39]. These aspects are increasingly central in data governance debates, especially in light of European regulatory developments such as the EHDS, which explicitly need to balance innovation, secondary use, public trust in health data ecosystems, and the use of AI tools such as large language models [19,20,24,40].

Research revealed that data donation via an opt-out regulation does not necessarily mean that the paradigm of informed consent has to be discontinued [23]. Instead, public education on data-intensive medical research and improved public “health data literacy” are needed [23]. Richter et al [24] discovered that German patients overwhelmingly supported data donation for medical research when “the combination of legal entitlement and easy-to-exercise-right to opt-out” was implemented. They recommended considering the incorporation of this approach into national law [24]. However, there is a great opportunity for networks and platforms to gain reputational benefits from conforming to rigorous data use standards such as the GDPR [41]. As previously reported, it is fundamental to implement and actively incorporate ethical, legal, and societal aspects of clinical and research data into governance structures and policies in research platforms and also to meet specifications for (local) institutional review boards [27,42,43]. Our platform established structural governance through, for example, a dedicated use and access board with ethics and data security experts.

Three broad consent items in our study referred to the donation of biomaterial-related data. Modern biobanks need to link biomaterials to secondary data sources, requiring funding and patients’ consent [44]. With a very high consent rate to these items, we showed that patients with IRDs are highly willing to connect their biomaterial-related information to secondary data sources. In addition, our agreement rates to broad consent items related to biomaterial data use were higher than those recently reported for the MII broad consent form [21]. This suggests that the biobank research community will be able to overcome challenges linking biomaterials to patient-related data in EHRs and other sources and reinforces our choice to include biomaterial data donation in the DNP scenario [44].

Crucially, the willingness to integrate biomaterial-linked omics and genomics data from prior donations further enhances the scientific and translational value of RWD and the DNP, enabling multimodal datasets that are essential for precision medicine approaches and AI-driven analytics, as well as for regulatory science, HTA, and other cross-border research purposes [27,39,45]. Although AI is not mentioned in depth in our current framework and the patient informed consent form, its relevance is substantial: platforms such as the DNP are inherently designed for longitudinal, high-dimensional, and multimodal data integration required for AI applications not only in rheumatology [46]. Consequently, our high broad consent agreement rates constitute a key enabler for scalable AI use in multifaceted health care research, including predictive modeling, patient stratification, and regulatory evidence generation.

Our findings on team time spent on broad consent align with the MII’s duration assessments on informed consent for secondary use of health care data and leftover biomaterials [47]. Our time spent informing patients was similar to the MII’s, and the time spent was regarded as helpful, informative, and sufficient [47]. Personal interaction with a study team member or neutral educator is seen as the most effective way to improve participants’ understanding [28], which likely contributed to our high recall and consent rates. Most of our patients kept their broad consent form copy, guaranteeing complete transparency and potential reassurance of what was signed. After 1 year, only 1 male patient with SLE withdrew consent, and patients asked for DNP results in routine care, reflecting sustained positive attitudes toward data donation to medical research and the DNP. However, the provision of broad consent– or study-related information to patients needs to be rethought as increasingly complex regulatory content requires innovative, multimedia-based approaches (eg, mixed reality and video clips) to convey information in an accessible, language-adapted, and comprehensible way [48]. Furthermore, while broad consent facilitates scalable use of data and biomaterials within a defined governance framework and dynamic consent enhances participant autonomy through ongoing control, neither model fully solves the structural limitations of traditional consent, highlighting a trade-off between efficiency and granularity that may be best addressed by hybrid approaches [27,49,50].

Recently, accessibility to RWD has improved at various levels, from national repositories to local hospital research databases [51]. Missing data and interoperability remain 2 of the most significant hurdles for RWD capture and use [10]. The European Alliance of Associations for Rheumatology core dataset agreements and European Reference Network efforts may help mitigate this [52-54]. Other obstacles to RWD research include access to suitable data sources and the need for sharing [51]. Federated learning approaches have been discussed to be advantageous over centralized databases (eg, in cancer, where RWD research is more advanced, or in the implementation of the EHDS [10,51,55,56]). European Union–funded projects develop big data infrastructures enabling large-scale data and AI integration [57]. These RWD infrastructures and other SPEs such as the DNP may link claims data with RWD for analyses. Initial studies, predominantly outside rheumatology, indicate that linking claims and registry data is feasible and useful [58-60]. Future approaches must consider that valuable EHR content will be revolutionized with the development of AI-driven data extraction and use [61]. With these ongoing developments, our patients’ extensive support for integrating patient-level RWD across different health care silos and settings provides a strong foundation to perform clinical and scientific research, and also in collaboration with the pharmaceutical industry and/or medical device manufacturers.

With the DNP, we established a firm legal basis for patient RWD integration rooted in informed consent pursuant to the GDPR, including key elements such as biobanked biomaterial data for research and permission for future use of modern AI methods.

### Strengths and Limitations

Despite a high response rate, selection and nonresponse bias cannot be excluded. Most participants were female patients with IRDs at a tertiary center; studies beyond such centers are needed. The observed high level of broad consent acceptance may represent an upper bound of patient willingness to participate in secondary data use. Consequently, the findings may not be directly generalizable to less engaged populations or different health care contexts, such as primary care settings or other routine clinical environments. The time spent obtaining broad consent in this study might not be feasible in routine care without reimbursement. Further research should explore data sharing willingness (eg, in general practices or private rheumatology practices); reasons for consenting, nonconsenting, or withholding consent to specific broad consent items (eg, via a qualitative approach); and understanding of content. Sex differences in completion rates require study in more balanced female-to-male ratios. The lowest consent rate was observed for informing other treating physicians about DNP participation, but our analyses did not reveal the reasons. Possible reasons might be complex phrasing or fatigue when nearing completion of the broad consent form. We did not specifically evaluate whether the involvement of commercial or private partners affected consent decisions. However, as study participation refusal was not communicated to be content related and no participants had reviewed the consent form prior to active refusal, this factor is, from the current perspective, unlikely to have meaningfully influenced participation in this study. The classification of patients into 2 groups was used as a conceptual framework to illustrate potential patterns of engagement with the broad consent form; however, patient behavior likely exists along a continuum rather than in discrete groups. Further research on these aspects is warranted.

### Conclusions

This study is the first to assess the willingness of patients with IRDs to grant broad consent for the secondary use of RWD within an innovative RWD platform that implements a modern framework in routine care while considering granular patient preferences. We found a high willingness among patients with IRDs to consent to the aims of the DNP. Our findings provide important RWE on actual consent behavior and deliver key insights for the implementation of scalable RWD integration initiatives, such as those envisioned within the EHDS and other data-driven research infrastructures. Furthermore, the DNP enables scalable, GDPR-compliant data sharing that supports real-world and AI-driven research, strategic decision-making, and the preservation of patient trust. We demonstrated that, in rheumatology, RWD use for RWE generation is not substantially constrained by patients’ willingness to provide broad consent.

## Abbreviations

AI: artificial intelligence
DNP: Data Nexus platform
EHDS: European Health Data Space
EHR: electronic health record
EMR: electronic medical record
GDPR: General Data Protection Regulation
HTA: health technology assessment
IRD: inflammatory rheumatic disease
IRD: inflammatory rheumatic disease
MII: Medical Informatics Initiative
PsA: psoriatic arthritis
RA: rheumatoid arthritis
RCT: randomized controlled trial
RWD: real-world data
RWE: real-world evidence
SLE: systemic lupus erythematosus
SpA: spondyloarthritis
SPE: secure processing environment
